# Exogenous Arginine Treatment Maintains the Appearance and Nutraceutical Properties of Hard- and Soft-Seed Pomegranates in Cold Storage

**DOI:** 10.3389/fnut.2022.828946

**Published:** 2022-05-19

**Authors:** Jiangli Shi, Huifang Gao, Sa Wang, Wenjiang Wu, Ruiran Tong, Sen Wang, Ming Li, Zaihai Jian, Ran Wan, Qingxia Hu, Xianbo Zheng, Yanhui Chen

**Affiliations:** ^1^College of Horticulture, Henan Agricultural University, Zhengzhou, China; ^2^Henan Key Laboratory of Fruit and Cucurbit Biology, Zhengzhou, China; ^3^College of Food Science and Technology, Henan Agricultural University, Zhengzhou, China

**Keywords:** pomegranate, arginine treatment, cold storage, chilling injury (CI), nutraceutical property

## Abstract

Arginine is a natural preservative; however, its effects on the storage of different cultivars of pomegranates have not been investigated extensively. Therefore, the fruit quality of soft-seed Tunisia and hard-seed Yudazi pomegranates was investigated after treatment with arginine at four concentrations during cold storage for 80 days. Pomegranates treated with 1.0 mM arginine exhibited a relatively lower loss of vitamin C, soluble solid, total phenol, and anthocyanin contents in arils, together with a better fruit appearance. Combined with principal component analysis (PCA), the storage life of fruits treated with 1.0 mM arginine showed a higher correlation with antioxidant enzyme activity (e.g., superoxide dismutase (SOD), ascorbate peroxidase (APX), and catalase (CAT)) during the first 40 days of cold storage, whereas after 40 days of cold storage, storage life was more dependent on the integrity of the cell membrane affected by malondialdehyde (MDA) content, electrolyte leakage (EL), and hydrogen peroxide (H_2_O_2_) accumulation. Arginine treatment contributed significantly to the appearance and inner quality of the hard-seed pomegranate cv. Yudazi fruit during cold storage compared to those of soft-seed Tunisia. Taken together, arginine application combined with cold storage enhanced the nutraceutical properties and marketability of pomegranate fruits.

## Introduction

*Punica granatum* L. (pomegranate) is an ancient medicinal fruit crop (1) that is grown commercially in China, India, Iran, Turkey, the United States, and Spain among other countries (2). Pomegranate is regarded as a “miracle fruit” and is popular owing to its high nutritional value and medicinal uses (1, 3–5). Generally, fruit quality and consumption trends depend on the appearance and inner quality changes. To meet the market demand for high quality and commercial value, cold storage is widely used to preserve fruits in combination with various treatments. However, pomegranate fruit consumption and processing are largely restricted by the harvesting season because of the high demand and lack of appropriate postharvest handling practices (6). Moreover, the loss of fruit quality frequently occurs during cold storage, and husk damage and physiological disorders severely affect the storage life and marketability of pomegranates, especially chilling injury and browning (7, 8).

Various treatments have been investigated to improve postharvest pomegranate quality, including controlled atmosphere (7, 9), shrink film wrap (10), heat treatment (11), and treatment with salicylic acid (12), methyl jasmonate (13), chitosan (14), and melatonin (15, 16). Arginine is an essential precursor in the synthesis of biologically important metabolites, such as polyamines and nitric oxide (17), and plays an important role in plant biotic and abiotic stresses, *Botrytis cinerea* infection (18), salt stress (19), and chilling injury (20–22). Furthermore, L-arginine derived from animal-based food or plant extracts functions as a natural preservative (23). Increasing evidence has shown that the use of L-arginine is safe and effective; therefore, arginine has been used in recent years to improve the nutritional properties and extend the storage life of horticultural crops, such as strawberry (17), asparagus (23), apple and lettuce (24), mushroom (25), peach (26), and broccoli (17). However, data on exogenous arginine application to pomegranate remain limited.

The differences in morphological and chemical characteristics among pomegranate cultivars can be affected by genetic backgrounds. Different pomegranate cultivars vary in their tolerance to low temperatures. Based on major differences between the two pomegranate cultivars, i.e., the soft-seed pomegranate cv. Tunisia showing low cold tolerance and hard-seed pomegranate cv. Yudazi showing high cold tolerance, the current study investigated the mechanisms *via* which arginine treatment alleviates postharvest chilling injury and maintains high fruit quality in the two cultivars, which may contribute to arginine application in a reliable manner.

## Materials and Methods

### Fruit Sample Preparation and Treatments

*Punica granatum* cvs. Tunisia and Yudazi trees were cultivated in commercial orchards in Xingyang, Henan, and China that have a warm temperate continental climate with four distinct seasons. Pomegranate cv. soft-seed Tunisia, introduced from Tunisia, had a low cold tolerance, whereas cv. hard-seed Yudazi, originally from China, had a stronger cold tolerance. The undamaged and healthy fruits (without sunburn, cracks, bruises, and cuts in the husk) of uniform size and appearance were harvested at the commercial maturity stage in 2018, namely, Tunisia (380 ± 30 g) on October 4 and Yudazi (320 ± 30 g) on October 16. The fruits were transported to the laboratory at Henan Agricultural University, Zhengzhou, Henan, China, and randomly assigned to five lots treated with different concentrations of arginine (0, 0.5, 1.0, 1.5, and 2.0 mM; Solarbio Life Sciences, Beijing, China); 0 mM treatment was the control (CK).

The pomegranate fruits were immersed in arginine solutions of four concentrations for 15 min, dried naturally, and stored in a controlled temperature chamber in permanent darkness at 4 ± 0.5°C with a relative humidity of 85%. The 10 tested fruits were sampled at 0, 20, 40, 60, and 80 days of storage for each treatment. The arils were manually separated and the inner white parts of the fruit were removed.

### Determination of Browning Rate, Electrolyte Leakage, and Malondialdehyde Content in Husks

The browning rate was visually evaluated based on the area of the husk surface affected by browning symptoms (e.g., dehydration, browning, and pitting). The electrolyte leakage (EL) rate was determined according to the method described by Mirdehghan et al. (11). Malondialdehyde (MDA) content was measured *via* the thiobarbituric acid method using the MDA-2-Y Kit (Suzhou Comin Biotechnology, Suzhou, China). The MDA content was expressed as μmol/kg of fresh weight (FW).

### Antioxidant Enzyme Activity in Husks and Arils

Superoxide dismutase (SOD) activity was determined by measuring the inhibition of photochemical reduction of nitro blue tetrazolium chloride (NBT) using the SOD-2-Y Kit (Suzhou Comin Biotechnology). One unit of SOD activity was defined as the amount of enzyme that catalyzed a 50% inhibition in NBT reduction. Catalase (CAT) activity was measured using the potassium permanganate titration method, and one unit of CAT activity was defined as a decrease in absorbance at 240 nm of 0.01 per min. One unit of ascorbate peroxidase (APX) activity was defined as the amount of enzyme that oxidized 1 μmol ascorbate/min.

### Polyphenol Oxidase and Phenylalanine Ammonia-Lyase Activities in Husks and Arils

Polyphenol oxidase (PPO) and phenylalanine ammonia-lyase (PAL) activities were determined using the method described by Nguyen et al. (27). PPO activity was determined based on the change in absorbance at 420 nm of 0.01 per min. PAL activity was defined as a change in absorbance at 290 nm of 0.01 units/h. PPO and PAL enzyme activities are expressed as mkat/kg of FW.

### Determination of H_2_O_2_ Content and 2,2-Diphenyl-1-Picrylhydrazyl Scavenging Activity

Hydrogen peroxide (H_2_O_2_) content in husks and arils was assayed according to Babalar et al. (21) and expressed on an FW basis in mmol/kg according to a standard curve. The 2,2-diphenyl-1-picrylhydrazyl (DPPH) free radical-scavenging activity was measured in husks, as described by Nakajima et al. (28). The reduction in DPPH (%) was calculated according to the following formula: Inhibition of DPPH (%) = (Abs control − Abs sample)/Abs control × 100, where Abs is the absorbance.

### Determination of Aril Quality Parameters

The soluble solids content (SSC) in pomegranate arils was analyzed using a WY060T handhold refractometer (Chengdu Qingyang Huarui Optical Instrument Factory, Sichuan, China), and values were presented as Brix degrees. Ascorbic acid in the arils was analyzed using the 2,6-dichlorophenol indophenol titration method. The total anthocyanin content was determined using the pH differential method. About 0.5 ml of pomegranate juice has measured the absorbance using an L9 UV-visible spectrophotometer (Shanghai Measuretech Instrument, Shanghai, China) at 510 and 700 nm in 4.5 ml buffers, 0.025 M KCl (pH 1.0), and 0.4 M CH_3_COONa (pH 4.5), respectively. The results are expressed as milligrams of cyanidin-3-glucoside per 1 L of pomegranate juice. The total phenol content was determined using the Folin–Ciocalteu method. The absorbance was measured at 725 nm using a spectrophotometer. A standard curve was generated using gallic acid to express the total phenol content as gallic acid equivalents (GAEs) mg/100 g of FW.

### Statistical Analysis

Principal component analysis (PCA) and one-way ANOVA were performed using the SPSS Statistics version 20 (IBM, Chicago, IL, United States). Significant differences were evaluated using one-way ANOVA and Duncan’s multiple range test. Different letters indicate significant differences among concentration treatments and sampling dates during cold storage (P < 0.05). The figures were created using Origin 8.0. All investigated parameters were measured on sampling days in triplicate.

## Results

### Effect of Arginine Treatment on Pomegranate Husks During Cold Storage

**FIGURE 1:**
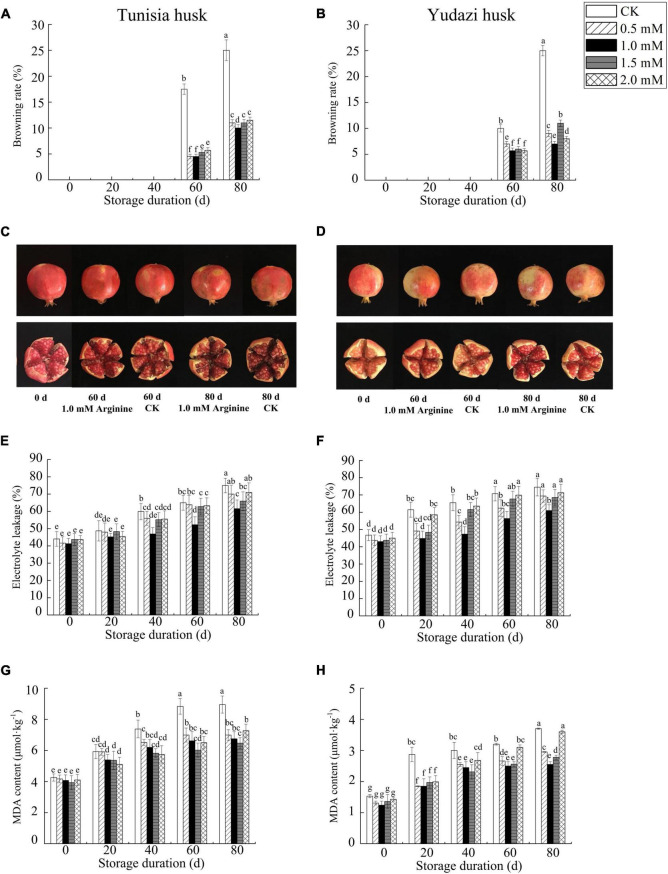
Effect of arginine treatment on browning rate, electrolyte leakage, and MDA content in pomegranate husks during cold storage at 4 ± 0.5°C. Panels **(A,C,E,G)** present Tunisia fruit data. Panels **(B,D,F,H)** present Yudazi fruit data. Values are presented as the mean ± standard error of triplicate samples. Different letters indicate significant differences among concentration treatments and sampling dates during the cold storage at *P* < 0.05.

No decay symptoms were found in the CKs and arginine-treated pomegranates during storage (Figures 1A–D). In addition, no trace levels of browning were observed during the first 40 days of storage. At 60 and 80 days after cold storage, the husks of both cultivars developed browning symptoms, and the intensity increased with the storage duration. The husk browning rate of the two cultivars was significantly different (*P* < 0.05) between arginine-treated fruits and their CKs, respectively (Figures 1A,B). The lowest husk browning rate was observed in fruits treated with 1.0 mM arginine. The browning rate of arginine-treated fruits was 4.5–11%, whereas that of the CKs reached 25% at 80 days of cold storage. Furthermore, the husk browning rate was lower in Tunisia fruits on the 60th day after cold storage than that in arginine-treated Yudazi fruits; however, it increased faster and was higher than that of the Yudazi fruits at 80 days of storage at 4°C (Figures 1A,B). Yudazi fruits exhibited relatively lower husk browning rates and arils of lighter shades of red (Figures 1C,D).

The EL and MDA accumulation is widely investigated to evaluate membrane integrity. The relative EL and MDA content in both cultivars showed an upward trend to a varying extent (Figures 1E–H). The husks after arginine treatments showed relatively lower EL and MDA accumulation during cold storage. Moreover, Yudazi husks treated with 1.0 mM arginine had a relatively lower EL and MDA accumulation from 20 days to 80 days, with a significant difference compared with those of CKs. In addition, Tunisia fruits had a higher MDA content than that in Yudazi fruits at all time points (Figures 1G,H), partially explaining the higher tolerance of hard-seed Yudazi to chilling stress than that of soft-seed Tunisia.

### Effect of Arginine on Antioxidant Enzyme Activity in Arils and Husks

The effect of arginine treatment on antioxidant enzyme activity in harvested pomegranate fruits was investigated during cold storage at 4°C. The SOD enzyme activity after arginine treatment in both arils and husks peaked at 20 days after cold storage, whereas that of the CKs in arils of the two cultivars decreased gradually during cold storage and reached the highest in husks at 20 days after cold storage (Tables 1 and 2). Furthermore, SOD activity was higher in Yudazi fruits than in Tunisia fruits and higher in husks than in arils. Notably, arils treated with 1.5 mM arginine and husks treated with 1.0 mM arginine showed relatively higher SOD activity. CAT and APX activities in arils and husks decreased during cold storage (Tunisia, shown in Table 1; Yudazi, shown in Table 2). However, treatment with 1.0 mM arginine slightly increased CAT and APX activities for the first 20 days after cold storage, which then declined in Tunisia and Yudazi pomegranates. At the beginning of the cold storage, APX activities in Tunisia husks and arils were considerably higher, but reduced quickly with storage time, especially in husks. Arginine treatment effectively inhibited the increase in H_2_O_2_ level at low temperatures compared with those in the CKs. The 1.0 mM treatment effectively reduced H_2_O_2_ accumulation in husks and arils of the two cultivars during cold storage (Tunisia, shown in Table 1; Yudazi, shown in Table 2).

**TABLE 1:** Enzyme activities of SOD, CAT, APX, and H_2_O_2_ content in Tunisia husks and arils during cold storage at 4 ± 0.5°C.

Parameters	Arginine treatment (mM)	Storage days				
		0	20	40	60	80
Aril SOD (mkat⋅kg^–1^)	0	0.72 ± 0.03b	0.69 ± 0.06bc	0.55 ± 0.03d	0.45 ± 0.04e	0.42 ± 0.05e
	0.5	0.74 ± 0.07b	0.75 ± 0.04b	0.56 ± 0.05cd	0.51 ± 0.05d	0.44 ± 0.05e
	1.0	0.79 ± 0.04ab	0.88 ± 0.05a	0.66 ± 0.06c	0.57 ± 0.05cd	0.55 ± 0.05d
	1.5	0.83 ± 0.07ab	0.88 ± 0.04a	0.68 ± 0.03c	0.61 ± 0.06c	0.59 ± 0.06cd
	2.0	0.77 ± 0.08ab	0.81 ± 0.07ab	0.64 ± 0.02c	0.59 ± 0.04cd	0.50 ± 0.04de
Husk SOD (mkat⋅kg^–1^)	0	1.77 ± 0.10bc	1.84 ± 0.11b	1.79 ± 0.10bc	1.70 ± 0.07c	1.53 ± 0.06d
	0.5	1.79 ± 0.10bc	1.94 ± 0.10ab	1.80 ± 0.06b	1.72 ± 0.06c	1.58 ± 0.05d
	1.0	1.90 ± 0.07b	2.08 ± 0.08a	1.93 ± 0.10ab	1.87 ± 0.09b	1.79 ± 0.06c
	1.5	1.86 ± 0.06b	2.04 ± 0.10a	1.84 ± 0.11b	1.73 ± 0.05c	1.66 ± 0.07c
	2.0	1.83 ± 0.07b	2.01 ± 0.08a	1.83 ± 0.08b	1.66 ± 0.05c	1.59 ± 0.05d
Aril CAT (g⋅(kg⋅min)^–1^)	0	22.80 ± 0.96a	21.72 ± 0.78b	20.88 ± 0.65bc	20.16 ± 0.78c	17.16 ± 0.50d
	0.5	22.80 ± 0.92a	21.96 ± 0.50b	19.80 ± 0.66d	19.44 ± 0.55c	17.40 ± 0.50d
	1.0	23.88 ± 1.04a	23.94 ± 0.42a	21.96 ± 0.55b	21.48 ± 0.60b	19.92 ± 0.44c
	1.5	23.52 ± 0.60a	21.72 ± 0.71b	21.00 ± 0.70bc	20.88 ± 0.75bc	19.56 ± 0.72c
	2.0	23.76 ± 0.84a	23.28 ± 0.64a	20.64 ± 0.56bc	19.92 ± 0.81c	17.40 ± 0.55d
Husk CAT (g⋅(kg⋅min)^–1^)	0	19.92 ± 0.80a	16.20 ± 0.70d	15.84 ± 0.26d	15.36 ± 0.50de	14.40 ± 0.40e
	0.5	19.15 ± 0.56a	16.80 ± 0.44d	14.52 ± 0.81e	13.56 ± 0.38f	13.08 ± 0.32f
	1.0	20.64 ± 0.68a	20.72 ± 0.61a	17.28 ± 0.66cd	17.16 ± 0.84cd	16.68 ± 0.72d
	1.5	20.88 ± 0.50a	20.28 ± 0.75a	18.96 ± 0.45b	17.40 ± 1.00cd	16.32 ± 0.46d
	2.0	19.20 ± 1.02a	16.08 ± 0.70d	16.08 ± 0.92d	15.60 ± 0.58d	14.88 ± 0.35e
Aril APX (mkat⋅kg^–1^)	0	31.12 ± 1.04a	29.20 ± 0.99ab	27.68 ± 1.00bc	25.48 ± 0.84d	25.01 ± 0.68d
	0.5	31.32 ± 0.92a	28.93 ± 0.70b	27.80 ± 1.03bc	25.98 ± 0.60d	25.34 ± 0.71d
	1.0	31.39 ± 1.09a	31.81 ± 1.05a	29.27 ± 1.07ab	27.47 ± 0.93c	27.25 ± 0.75c
	1.5	31.68 ± 1.00a	29.87 ± 0.81ab	28.33 ± 1.09bc	26.58 ± 0.50cd	26.54 ± 0.71cd
	2.0	29.03 ± 0.90ab	28.03 ± 0.87bc	25.01 ± 1.02d	23.34 ± 0.78e	23.02 ± 0.65e
Husk APX (mkat⋅kg^–1^)	0	10.28 ± 0.52ab	9.24 ± 0.33b	5.47 ± 0.22d	3.33 ± 0.22f	2.26 ± 0.25g
	0.5	10.43 ± 0.45ab	7.86 ± 0.38c	5.61 ± 0.27d	4.44 ± 0.29e	3.09 ± 0.32f
	1.0	11.36 ± 0.36a	11.55 ± 0.39a	9.11 ± 0.30b	5.78 ± 0.20d	4.07 ± 0.19e
	1.5	10.76 ± 0.40a	7.21 ± 0.21c	6.94 ± 0.25c	6.43 ± 0.34cd	3.27 ± 0.37f
	2.0	10.83 ± 0.37a	8.67 ± 0.15b	6.67 ± 0.27c	3.86 ± 0.15ef	2.57 ± 0.30g
Aril H2O2 (mmol⋅kg^–1^)	0	99.89 ± 2.39g	114.44 ± 2.98f	127.41 ± 2.75de	138.30 ± 3.59cd	175.67 ± 5.13a
	0.5	92.14 ± 2.15g	99.37 ± 1.96g	113.07 ± 2.39f	126.96 ± 4.89de	151.56 ± 3.98b
	1.0	95.46 ± 4.19g	103.29 ± 4.48fg	106.16 ± 4.20f	115.41 ± 2.80ef	120.69 ± 4.59e
	1.5	93.11 ± 4.06g	106.31 ± 1.20fg	110.72 ± 4.78f	125.98 ± 1.96e	132.04 ± 4.67d
	2.0	92.00 ± 4.11g	105.05 ± 3.54fg	108.37 ± 2.61f	119.33 ± 4.87e	143.00 ± 4.00c
Husk H2O2 (mmol⋅kg^–1^)	0	109.96 ± 2.76e	120.55 ± 5.30d	132.85 ± 3.12cd	160.29 ± 1.37b	199.92 ± 6.82a
	0.5	107.05 ± 4.54e	115.90 ± 3.52de	128.94 ± 5.08d	147.58 ± 2.35c	193.57 ± 6.39a
	1.0	101.46 ± 3.13e	114.63 ± 3.50de	120.85 ± 4.78d	123.26 ± 0.98d	137.37 ± 4.56c
	1.5	102.74 ± 5.24e	115.87 ± 5.00de	124.42 ± 5.10d	129.90 ± 5.30d	143.19 ± 3.90c
	2.0	106.83 ± 3.41e	108.24 ± 4.50e	133.29 ± 5.04cd	160.19 ± 5.00b	189.95 ± 6.06a

*Values are presented as the mean ± standard error (n = 3). Different letters indicate significant differences among concentration treatments and sampling dates during cold storage (P < 0.05). SOD, superoxide dismutase; APX, ascorbate peroxidase; CAT, catalase; H_2_O_2_, hydrogen peroxide.*

**TABLE 2:** Enzyme activities of SOD, CAT, APX, and H_2_O_2_ content in Yudazi husks and arils during cold storage at 4 ± 0.5°C.

Parameters	Arginine treatment (mM)	Storage days				
		0	20	40	60	80
Aril SOD (mkat⋅kg^–1^)	0	0.89 ± 0.07b	0.89 ± 0.06b	0.78 ± 0.02c	0.53 ± 0.04e	0.44 ± 0.03f
	0.5	0.92 ± 0.08ab	0.96 ± 0.05ab	0.81 ± 0.03c	0.61 ± 0.04d	0.51 ± 0.04e
	1.0	0.96 ± 0.04ab	1.00 ± 0.06a	0.82 ± 0.05c	0.62 ± 0.04d	0.57 ± 0.04de
	1.5	0.95 ± 0.07ab	1.02 ± 0.06a	0.89 ± 0.07b	0.65 ± 0.03d	0.62 ± 0.04d
	2.0	0.91 ± 0.08ab	0.94 ± 0.07ab	0.80 ± 0.03c	0.55 ± 0.06de	0.46 ± 0.03f
Husk SOD (mkat⋅kg^–1^)	0	2.67 ± 0.10c	2.79 ± 0.10c	2.74 ± 0.09c	2.60 ± 0.08d	2.43 ± 0.06e
	0.5	2.68 ± 0.08c	3.18 ± 0.10b	2.92 ± 0.09bc	2.80 ± 0.09c	2.49 ± 0.10e
	1.0	2.76 ± 0.11c	3.46 ± 0.11a	3.05 ± 0.10b	2.93 ± 0.08bc	2.66 ± 0.09d
	1.5	2.71 ± 0.13c	3.37 ± 0.09a	3.03 ± 0.11b	2.97 ± 0.10b	2.57 ± 0.15d
	2.0	2.70 ± 0.10c	3.24 ± 0.15a	3.01 ± 0.13b	2.98 ± 0.09b	2.56 ± 0.13d
Aril CAT (g⋅(kg⋅min)^–1^)	0	22.32 ± 0.48ab	21.12 ± 0.46bc	20.64 ± 0.50c	19.92 ± 0.85cd	18.72 ± 0.35d
	0.5	22.44 ± 0.36ab	21.24 ± 0.80bc	21.12 ± 0.70bc	20.64 ± 0.45c	19.92 ± 0.44cd
	1.0	22.92 ± 0.60a	23.80 ± 0.46a	22.68 ± 0.60a	21.48 ± 0.56b	21.24 ± 0.38c
	1.5	22.56 ± 0.90ab	22.20 ± 0.48b	21.60 ± 0.56b	20.76 ± 0.72bc	20.04 ± 0.50cd
	2.0	22.20 ± 0.75ab	20.64 ± 0.72c	20.88 ± 0.50bc	19.92 ± 0.55cd	19.68 ± 0.62cd
Husk CAT (g⋅(kg⋅min)^–1^)	0	22.56 ± 0.48ab	21.24 ± 0.55b	20.64 ± 0.60c	20.04 ± 0.62d	18.60 ± 0.48e
	0.5	22.56 ± 0.40ab	20.40 ± 0.50c	19.08 ± 0.25d	18.60 ± 0.35e	18.12 ± 0.50e
	1.0	23.52 ± 0.54a	23.86 ± 0.45a	21.48 ± 0.48b	20.64 ± 0.30bc	20.40 ± 0.45c
	1.5	23.42 ± 0.76a	20.88 ± 0.72bc	20.64 ± 0.78bc	20.40 ± 0.39c	20.16 ± 0.52cd
	2.0	23.40 ± 0.52a	21.36 ± 0.60b	20.28 ± 0.56cd	20.04 ± 0.42c	19.44 ± 0.49de
Aril APX (mkat⋅kg^–1^)	0	15.09 ± 0.63ab	13.34 ± 0.51bc	12.08 ± 0.56de	10.76 ± 0.33ef	9.56 ± 0.31g
	0.5	14.90 ± 0.51ab	13.34 ± 0.66bc	11.52 ± 0.30e	10.73 ± 0.38ef	9.91 ± 0.42g
	1.0	15.94 ± 0.60a	16.06 ± 0.45a	12.84 ± 0.30d	11.34 ± 0.26e	11.18 ± 0.25e
	1.5	15.84 ± 0.48a	14.10 ± 0.46b	12.93 ± 0.37d	11.15 ± 0.33e	10.84 ± 0.37ef
	2.0	15.43 ± 0.41ab	13.84 ± 0.40c	12.42 ± 0.56d	11.35 ± 0.30e	10.51 ± 0.50efg
Husk APX (mkat⋅kg^–1^)	0	2.81 ± 0.10bc	2.28 ± 0.11e	2.24 ± 0.08e	2.08 ± 0.12f	1.85 ± 0.08g
	0.5	3.00 ± 0.11b	2.50 ± 0.12cd	2.40 ± 0.08d	2.33 ± 0.11de	2.10 ± 0.14f
	1.0	3.04 ± 0.10b	3.33 ± 0.10a	2.78 ± 0.06c	2.72 ± 0.09c	2.62 ± 0.10c
	1.5	2.83 ± 0.13bc	2.58 ± 0.13cd	2.43 ± 0.05d	2.30 ± 0.13de	2.25 ± 0.11e
	2.0	2.89 ± 0.10bc	2.67 ± 0.11c	2.39 ± 0.10d	2.34 ± 0.10de	2.23 ± 0.07e
Aril H2O2 (mmol⋅kg^–1^)	0	74.20 ± 6.26e	89.40 ± 7.80cd	103.48 ± 1.37c	115.81 ± 7.00b	128.94 ± 0.39a
	0.5	68.60 ± 6.21e	89.66 ± 0.20d	100.58 ± 5.69c	105.24 ± 7.22bc	116.68 ± 5.91b
	1.0	67.31 ± 0.98e	88.81 ± 0.78cd	92.85 ± 5.78d	93.55 ± 1.56d	98.09 ± 2.93c
	1.5	67.50 ± 5.74e	91.74 ± 5.24cd	95.82 ± 7.80c	101.27 ± 5.28c	112.48 ± 0.20b
	2.0	68.00 ± 0.75e	86.83 ± 7.41cd	98.98 ± 4.69c	110.05 ± 5.06bc	114.72 ± 7.43b
Husk H2O2 (mmol⋅kg^–1^)	0	101.00 ± 6.06d	116.81 ± 1.96c	118.92 ± 3.60bc	120.11 ± 8.61bc	138.61 ± 3.59a
	0.5	100.00 ± 5.51d	109.05 ± 7.24c	112.67 ± 5.78c	115.35 ± 7.50bc	127.20 ± 3.91b
	1.0	92.92 ± 3.50d	109.74 ± 5.67c	112.85 ± 5.75c	114.15 ± 5.22c	115.39 ± 5.20c
	1.5	94.74 ± 6.24d	101.44 ± 3.33d	114.26 ± 1.76c	115.41 ± 1.96c	120.15 ± 2.35c
	2.0	96.83 ± 5.41d	105.79 ± 7.10cd	109.90 ± 4.80c	114.48 ± 7.04bc	125.31 ± 3.90b

*SOD, superoxide dismutase; APX, ascorbate peroxidase; CAT, catalase; H_2_O_2_, hydrogen peroxide.*

### Effect of Arginine Treatment on PAL and PPO Enzyme Activities in Arils and Husks

**FIGURE 2:**
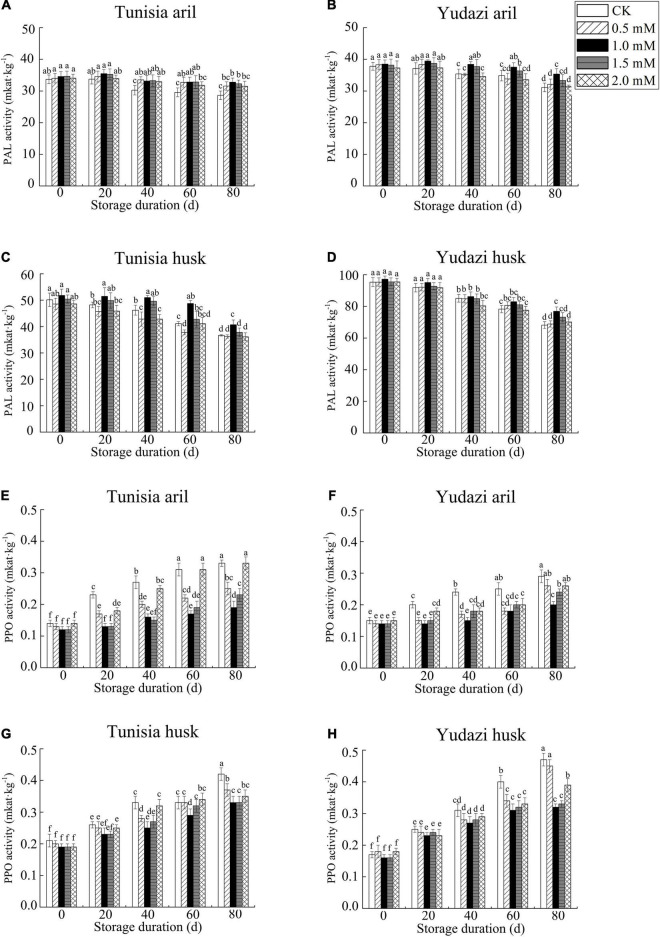
Effect of arginine treatment on PAL activity and PPO activity in arils and husks from the two cultivars during cold storage at 4 ± 0.5°C. Panels **(A,C,E,G)** present Tunisia. Panels **(B,D,F,H)** present Yudazi. Different letters present significant differences among concentration treatments and sampling dates during the cold storage at *P* < 0.05.

The PAL enzyme activity declined in the two cultivars during the 80 days of cold storage (Figures 2A–D). However, the highest activity in the arils was observed at 20 days of cold storage. PPO enzyme activity in the two cultivars showed an increasing trend with storage duration (Figures 2E–H). The increase in PPO enzyme activity after 1.0 mM arginine treatment was slower than the increase after treatment with 0.5, 1.5, and 2.0 mM arginine. The longer the storage duration, the more significant the difference was (P < 0.05).

### Components and Nutritional Value in Pomegranate Arils During Cold Storage

**FIGURE 3:**
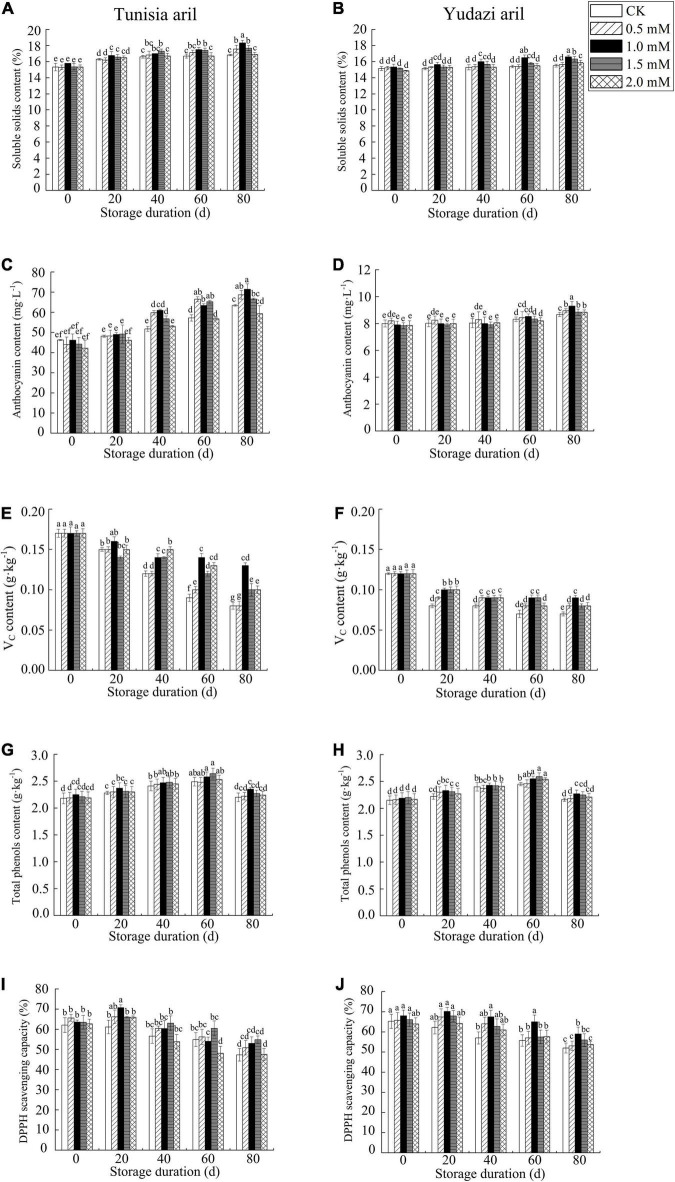
Contents of SSC, anthocyanin, vitamin C, total phenols, and DPPH radical-scavenging capacity in pomegranate arils after arginine treatments during storage at 4 ± 0.5°C. Panels **(A,C,E,G,I)** present Tunisia. Panels **(B,D,F,H,J)** present Yudazi. Values are mean ± standard error of triplicate samples. Different letters showed significant differences among concentration treatments and sampling dates during the cold storage at *P* < 0.05. SSC, soluble solids content; DPPH, 2,2-diphenyl-1-picrylhydrazyl.

The SSC in arils increased slightly with storage duration, and 1.0 mM arginine treatment showed a significant difference in SSC between the CK groups at 60 and 80 days of storage (Figures 3A,B). Anthocyanins are important phenolic compounds present in pomegranates that also affect the color of fruits. To verify the effect of arginine on postharvest pomegranate fruits during cold storage, the anthocyanin content in the arils was investigated in the current study. Higher anthocyanin and vitamin C contents were found in Tunisia fruit arils than those in Yudazi fruit arils, and arginine treatment reduced the loss in anthocyanin content (Figures 3C–F). In contrast, with increasing anthocyanin content, the vitamin C content in pomegranate arils declined with the storage period (Figures 3E,F). The total phenol content in the arils peaked at 60 days and then declined during cold storage (Figures 3G,H). The total phenol contents in Tunisia and Yudazi fruits treated with 1.0 mM arginine were significantly different from those in their CK groups until 80 days of cold storage. The DPPH radical-scavenging activity in the arginine treatment groups was higher than that in the CK group during cold storage, except at 2.0 mM arginine treatment (Figures 3I,J).

### Principal Component Analysis

**FIGURE 4:**
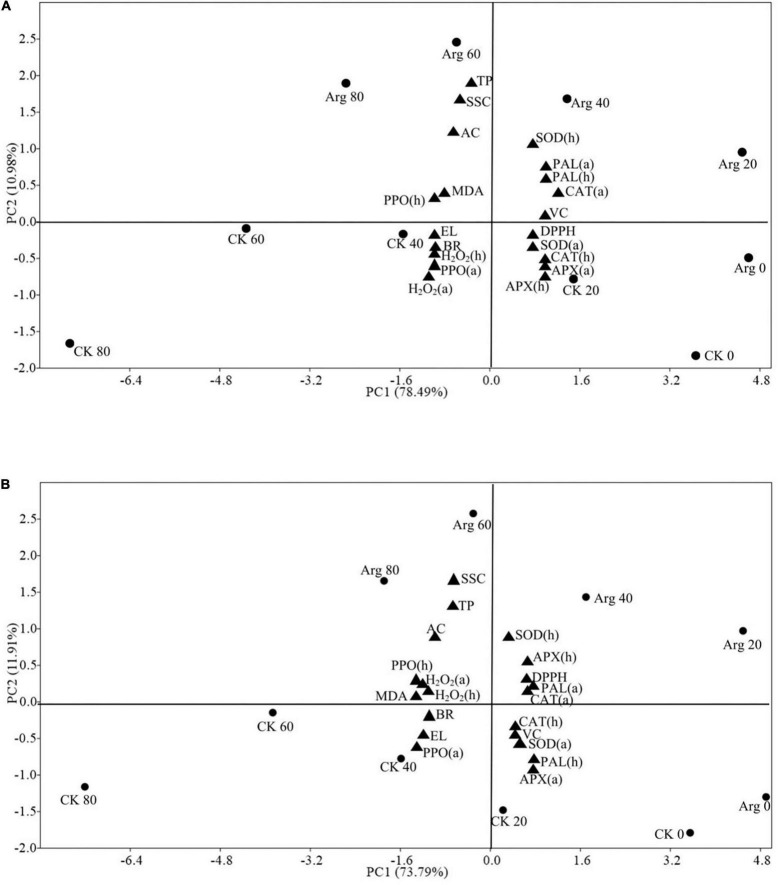
PCA plot of fruit qualities and antioxidant activities in **(A)** Tunisia and **(B)** Yudazi pomegranate (a) arils and (h) husks treated with 1.0 mM Arg at 0, 20, 40, 60, and 80 days of the cold storage at 4 ± 0.5°C. BR, browning rate; EL, electrolyte leakage; AC, anthocyanin content; VC, vitamin C content; TP, total phenols content; Arg, arginine; CK, control fruit.

The PCA of two-dimensional scatter plots was performed *via* the evaluation of physicochemical properties and antioxidant enzyme activities in pomegranates treated with 1.0 mM arginine during cold storage (Figure 4). PC1 and PC2 accounted for 78.49% and 10.98% of the total variation in Tunisia fruits (Figure 4A) and 73.79% and 11.91% of the total variance in Yudazi fruits (Figure 4B), respectively. The maximum possible variation during cold storage was explained by PC1. The two cultivars displayed similar results. Positive scores on the right-side plot of PC1 axis correspond to the antioxidant enzyme activity, including SOD, APX, CAT, PAL, and DPPH scavenging capacity, and Vc content; the first 40 days with arginine treatment and the first 20 days without arginine treatment during cold storage were also associated with positive scores of PC1 axis. However, EL, the browning rate, and PPO in arils were clustered on the opposite side of PC1 and PC2 axes (Figure 4). Treatment with 1.0 mM arginine can prolong the storage time of pomegranate fruits by enhancing their antioxidant enzyme activity. The SSC, total phenol, and anthocyanin contents, and the browning rate, PPO activity, and MDA and H_2_O_2_ contents were negatively correlated with the PC1 axis and corresponded to 60 and 80 days with arginine treatment and 40, 60, and 80 days without arginine treatment during cold storage (Figure 4).

## Discussion

Cold storage is widely used to maintain the nutritional value of fruits and vegetables. However, low temperatures are likely to cause chilling injury (8). Treatment with polyamines (e.g., putrescine, spermidine, and spermine), proline, and nitric oxide can improve the quality and prolong the storage life of fruits, such as apricot (29), kiwi (30), mango (31, 32), strawberry (33), loquat (34), and pomegranate (20, 35). This study showed that the storage life of pomegranate fruits treated with L-arginine was prolonged compared with that of CKs, and Yudazi fruits maintained a relatively lower browning rate and better fruit quality. The 1.0 mM arginine treatment achieved a better storage effect among the concentrations tested.

The browning of pomegranate husks represents a common problem during cold storage. Husk browning is generally attributed to chilling injury (11, 16). Therefore, cultivars that differed in tolerance to low temperatures were selected to further explore the effects of storage. Furthermore, both cultivars displayed obvious differences in husk and aril appearance during cold storage. Wills and Li (24) reported that arginine treatment inhibits surface browning on fresh-cut apples and lettuce. Mirdehghan et al. (20) observed that the application of 1 mM putrescine or spermidine significantly reduces skin browning in pomegranates. In our study, 1.0 mM arginine treatment was relatively more effective in inhibiting browning during cold storage, which was attributed to the reduction in EL and MDA accumulation in the husks, which maintained the integrity of the cell membrane, consistent with previous studies on pomegranates (20, 21). Notably, fruits treated with 1.0 mM arginine treatment were significantly different from CKs; the changes appeared after 20 days of cold storage for Yudazi fruits and after 40 days for Tunisia fruits, suggesting that the different responses to arginine were due to different cultivar traits.

Pomegranate juice represents a rich source of bioactive compounds that are beneficial to human health. In recent years, several studies have demonstrated significant differences in SSC, anthocyanin content, vitamin C content, and antioxidant enzyme activity among pomegranate cultivars or clones (36–38). We found that at the initial levels of anthocyanin content in arils, the content in Tunisia fruits (44.58 mg/L) was considerably higher than that in Yudazi fruits (7.96 mg/L). Moreover, the accumulation of anthocyanins and SSC in arils during cold storage was also reported by Selcuk and Erkan (9), Aghdam et al. (16), Mirdehghan et al. (20), and Amiri et al. (39), which may be attributed to the rapid moisture loss occurring in juicy fruits that increases with storage duration. Our findings demonstrated that arils treated with 1.0 mM arginine maintained the highest levels of anthocyanin and SSC at the end of storage, which was also confirmed in the PCA plot. Anthocyanin degradation in pomegranates may be delayed to a certain extent by treatments with exogenous arginine, melatonin (15, 16), methyl salicylate (13), and *via* ultrasonication (39). In addition, the decrease in Vc content in arils during cold storage was consistent with previous reports on the application of arginine to broccoli (17), strawberry (17), and pomegranate (21), and in putrescine-treated pomegranate cv. Mridula (35) and pomegranate cv. Hicaznar in modified atmosphere packaging (9). DPPH radical-scavenging activity in the arginine treatment groups was higher than in the CKs, except at 2.0 mM, suggesting that only a suitable arginine concentration could enhance DPPH radical-scavenging activity. Based on the aforementioned results, 1.0 mM arginine was the preferred concentration for preserving harvested pomegranates in cold storage because it maintained high aril quality by reducing nutritional loss (e.g., anthocyanins, vitamin C, and phenols) and improving DPPH scavenging activity in the two cultivars.

Previous studies on postharvest horticultural crops have shown that high levels of antioxidant enzymes, including SOD, CAT, and APX, can alleviate chilling injury accompanied by scavenging reactive oxygen species, thereby meeting the market demand for high antioxidant functions for human health (40). Our results showed that 1.0 mM arginine treatment significantly delayed the decrease in SOD, CAT, and APX activities, together with H_2_O_2_ accumulation in husks and arils at 80 days of cold storage, which was consistent with previous findings (21, 23). In summary, the increased husk browning rate in Tunisia pomegranates may result from the relatively higher H_2_O_2_ content and lower SOD and CAT activities, along with a rapid decrease in APX activity, which partially accounts for the better storage effect observed for Yudazi fruits. In addition, Yudazi fruits have relatively better cold tolerance during storage owing to their cultivation in northern China.

Generally, an increase in PAL activity is considered an environmental stress response in plants. PPO is the main enzyme that causes browning and can catalyze the oxidation of polyphenols in fruits and vegetables to form brown compounds (41). In the current study, the decrease in PAL activity and increase in PPO activity in husks and arils were delayed significantly after 1.0 mM arginine treatment at 80 days of cold storage, which was similar to the results reported by Babalar et al. (21), Li et al. (25), and Sohail et al. (17). Regarding the consistency in PPO activity and browning rate, arginine application decreased PPO activity and consequently alleviated browning in pomegranates, which was also proposed by Li et al. (25) who reported the application of exogenous arginine to button mushrooms.

The PCA plot showed that without arginine treatment, the MDA content, EL, H_2_O_2_ accumulation, and PPO activity were closely related to the browning rate. However, after 1 mM arginine treatment, the fruit quality was greatly affected by Vc content and antioxidant enzyme activities, including SOD, APX, CAT, and PAL activities, especially at 20 and 40 days. At a later storage time (60 and 80 days), the aril traits greatly depended on SSC, total phenol content, and anthocyanin content, partially resulting from water loss. Collectively, it was proposed that arginine treatment improved cold tolerance, which provides valuable information for the application of arginine in fruit storage. Therefore, arginine is a safe natural preservative that is beneficial for prolonging the storage life of pomegranates.

## Conclusion

This study showed that a suitable arginine concentration in combination with cold storage represents an effective approach to maintain fruit quality and prolonging storage life, including soft-seed and hard-seed pomegranate cultivars. Combined with PCA, higher MDA content and EL reduced the integrity of the cell membrane, which accelerated browning due to relatively higher PPO enzyme activity and H_2_O_2_ accumulation. However, arginine treatment delayed this increase. Moreover, among the four concentrations tested, the optimum concentration was found to be 1.0 mM. Husk browning had a relatively higher correlation with H_2_O_2_ accumulation, together with antioxidant enzyme activity, including SOD, CAT, and APX activities. Although the anthocyanin, Vc, and total phenol contents, and APX activity in arils were higher in Tunisia fruits than in Yudazi fruits, the relatively higher CAT and SOD activities in Yudazi husks played a potential role in suppressing husk browning. Yudazi pomegranates are cultivated in northern China and have adapted to a low temperature, which may represent the reason for the better fruit quality of Yudazi during cold storage. In brief, the higher the tolerance of the cultivar to low temperature, the lower the loss of antioxidant enzyme capacity, and correspondingly, the longer the storage life in cold storage.

## Data Availability Statement

The original contributions presented in the study are included in the article/supplementary material, further inquiries can be directed to the corresponding author/s.

## Author Contributions

JS and XZ designed the research and revised the manuscript. HG, SaW, RT, and SeW performed experimental works and wrote the manuscript. WW and ML participated in data analysis. YC, RW, ZJ, and QH provided the technical support. All authors have read and agreed to the published version of the manuscript.

## Conflict of Interest

The authors declare that the research was conducted in the absence of any commercial or financial relationships that could be construed as a potential conflict of interest.

## Publisher’s Note

All claims expressed in this article are solely those of the authors and do not necessarily represent those of their affiliated organizations, or those of the publisher, the editors and the reviewers. Any product that may be evaluated in this article, or claim that may be made by its manufacturer, is not guaranteed or endorsed by the publisher.
